# Aplastic crisis and transient neurological symptoms due to parvovirus B19 infection in a patient with chronic hemolytic disorder

**DOI:** 10.1186/1471-2334-13-S1-P87

**Published:** 2013-12-16

**Authors:** Iulia Niculescu, Ana Maria Petrescu, Adriana Hristea, Victoria Aramă, Virgil Ionescu, Adrian Streinu-Cercel

**Affiliations:** 1 National Institute for Infectious Diseases “Prof. Dr. Matei Balş”, Bucharest, Romania; 2 Carol Davila University of Medicine and Pharmacy, Bucharest, Romania

## Background

Human parvovirus B19 is considered an important trigger of aplastic crisis in patients with chronic congenital hemolytic disorders.

## Case report

We describe the case of a young adult known with hereditary microspherocytosis who presented with fever, marked agitation and confusion and a slight left hemiparesis. The cerebral imaging revealed multiple giant cystic Virchow-Robin spaces, but no other abnormalities. The complete blood counts showed pancytopenia and the peripheral blood smear revealed reticulocytopenia, confirming the diagnosis of aplastic crisis. Human parvovirus B19 infection was proven by the detection of serum DNA using PCR technique. After the initiation of packed red cells transfusion a favorable outcome was seen and his neurologic symptoms fully remitted.

## Conclusion

Direct invasion of human parvovirus B19 may induce both aplastic crisis and acute encephalopathy in patients with hereditary spherocytosis. Human parvovirus B19 may induce aplastic crisis but also acute encephalopathy through direct invasion, although in our case the transient neurological symptoms were rather an effect of hypoxia.

